# A clinical practice-based evaluation of the RIETE score in predicting occult cancer in patients with venous thromboembolism

**DOI:** 10.1007/s11239-019-01822-z

**Published:** 2019-02-09

**Authors:** Axel Rosell, Staffan Lundström, Nigel Mackman, Håkan Wallén, Charlotte Thålin

**Affiliations:** 1 Department of Clinical Sciences, Danderyd Hospital, Division of Internal Medicine, Karolinska Institutet, Stockholm, Sweden; 2 Palliative Care Services and R&D-unit, Stockholms Sjukhem Foundation, Stockholm, Sweden; 3 Department of Oncology-Pathology, Karolinska Institutet, Stockholm, Sweden; 4 Department of Medicine, Division of Hematology and Oncology, Thrombosis and Hemostasis Program, University of North Carolina at Chapel Hill, Chapel Hill, NC USA; 5 Department of Clinical Sciences, Danderyd Hospital, Division of Cardiovascular Medicine, Karolinska Institutet, Stockholm, Sweden

**Keywords:** Venous thromboembolism, Occult cancer, Risk factor, Risk score, Screening strategy

## Abstract

The association between venous thromboembolism (VTE) and occult cancer is well established. However, the benefit of cancer screening in all VTE patients remains controversial. The Registro Informatizado Enfermedad TromboEmbólica (RIETE) score is a recently proposed risk score to identify VTE patients at high risk of occult cancer. We evaluated the performance of the RIETE score in a routine clinical setting comprising patients presenting with VTE between January 1 and December 31, 2014, at Danderyd University hospital. Out of 488 VTE patients, 47 (9.6%) patients received a new cancer diagnosis during a 24-month follow-up. After exclusion of patients with cancer diagnosed at baseline (≤ 10 days after VTE, n = 16), 472 patients were considered eligible for cancer screening. Among these 472 patients, 31 (6.6%) received a cancer diagnosis during follow-up. The cumulative incidence was high after both unprovoked (8.5%) and provoked (4.8%) VTE. The RIETE score was evaluated in 467 of these patients. Interestingly, a high RIETE score was not significantly associated with cancer diagnosis during follow-up (OR 1.78; 95% CI 0.85–3.63), which was mainly due to a poor performance in women (OR 1.04; 95% CI 0.30–2.83). In summary, we observed a relatively high incidence of occult cancer in both unprovoked and provoked VTE. The RIETE score performed poorly in identifying patients at high risk of occult cancer in our VTE population. Additional risk assessment models are warranted to identify VTE patients who would benefit from extensive cancer screening.

**Electronic supplementary material:**

The online version of this article (10.1007/s11239-019-01822-z) contains supplementary material, which is available to authorized users.

## Highlights

- The rate of occult cancer is elevated after both unprovoked and provoked VTE
- The RIETE score did not identify patients with a high risk of occult cancer in a routine clinical setting
- The low discriminative capacity of the RIETE score was mainly driven by a poor performance in women
- New risk score models are warranted to aid in identifying VTE patients at high risk of occult cancer

## Introduction

Venous thromboembolism (VTE), i.e. deep venous thrombosis (DVT) and pulmonary embolism (PE), is a common complication of cancer—the risk of VTE in cancer patients is four to sixfold compared to the general population [1, 2]. VTE may also be the earliest sign of occult cancer. Approximately 7–10% of patients with an unprovoked VTE (in the absence of a major risk factor) receive a new cancer diagnosis within 1 year [3, 4]. The incidence of occult cancer in patients with provoked VTE is less investigated, but is considered much lower [3]. However, the benefit and extent of screening for occult cancer in VTE patients remains controversial.

In theory, extensive screening of all patients with unprovoked VTE could lead to earlier detection of occult cancer, earlier initiation of anti-cancer treatment, and improved prognosis. However, recent prospective randomized trials have failed to show that extensive screening with computed tomography (CT) of abdomen/pelvis diagnose more cancers compared to a limited screening approach [5–7]. The addition of 18F-Fluorodesoxyglucose Positron Emission Tomography/Computed Tomography (FDG PET/CT) to limited diagnostic work-up has yielded promising results [8], but further studies are needed to clarify the benefit of FDG PET/CT in occult cancer screening.

To improve the identification of VTE patients who are at increased risk of occult cancer, and thus would benefit from a rapid and extensive cancer screening, a prediction score was recently developed from the Registro Informatizado Enfermedad TromboEmbólica (RIETE) registry database. The study included patients with unprovoked and provoked VTE, but only 12% of patients with no cancer at VTE presentation were included in the study [9]. Three validations of the RIETE score have been published [10–12]. These validations are, however, hampered by several limitations. The first validation was performed in a cohort of unprovoked VTE patients from the same registry that was used to develop the score and thus had a similar rate of missing data [10]. Post hoc analyses have also been performed on VTE patients included in two clinical trials, the MVTEP study (unprovoked VTE) [11] and the Hokusai-VTE trial (unprovoked and provoked VTE) [12]. Patients included in clinical trials are, however, generally healthier than those found in routine health care, which is a limitation of these studies. Therefore, our aim was to evaluate the performance of the RIETE score in identifying patients with a high risk of occult cancer in a clinical practice-based setting comprising patients with both provoked and unprovoked VTE.

## Methods

### Study design and study population

Patients diagnosed with DVT and/or PE (confirmed by ultrasonography, phlebography or CT angiography for DVT and helical CT scan or ventilation-perfusion lung scintigraphy for PE), at the departments of medicine and cardiology at Danderyd University hospital in Stockholm, Sweden, between January 1 and December 31, 2014, were identified and included in a retrospective cohort study. Patients under the age of 18 were excluded in accordance with the decision from the regional ethical review board. The hospital is a secondary referral center and has a catchment area of approximately 500,000 inhabitants. Patients were identified through a search in the hospital registries using ICD10-codes I80-82 and I26; medical records were examined individually to confirm the diagnosis of VTE. In case a patient received several VTE diagnoses during the inclusion period, only the first event was considered. Patients not residing in Stockholm county were excluded.

### Baseline variables

Baseline variables were obtained from medical records, and included sex, age, BMI, prior cancer, type of VTE (DVT, PE, or DVT + PE), hereditary thrombophilia, chronic obstructive pulmonary disease (COPD), smoking status, comorbidities (diabetes mellitus, prior ischemic stroke/transitory ischemic attack, prior myocardial infarction, heart failure) and prior VTE. Routine laboratory data, including hemoglobin levels, white blood cell count and platelet count, were extracted manually from medical records.

Provoking factors were adapted from the Scientific and Standardization Committee of the International Society of Thrombosis and Haemostasis [13]. Patients categorized with a provoked VTE had either a *major transient* risk factor during the 3 months before diagnosis of VTE (i.e. cesarean section, surgery with general anesthesia for more than 30 min, or confined to bed in hospital for at least 3 days with an acute illness), a *minor transient* risk factor during the 2 months before diagnosis of VTE (i.e. estrogen therapy, pregnancy, puerperium, surgery with general anesthesia for less than 30 min, admission to hospital for less than 3 days with an acute illness, leg injury associated with reduced mobility for at least 3 days and confined to bed out of hospital for at least 3 days [i.e. bedridden]), or a *persistent* risk factor (active cancer or a non-malignant condition associated with a > twofold risk increase of recurrent VTE after stopping anticoagulant therapy [13] [e.g. inflammatory disease]). Cancer was considered active if any of the following applied: (1) no potentially curative anti-cancer therapy given; or (2) evidence of recurrent or progressive disease, or (3) ongoing anti-cancer therapy [13]. For ease of presentation and sub-analyses, surgical provoking factors and hospital stay provoking factors were combined. In addition to the provoking factors proposed by ISTH, we also considered long distance travel > 6 h during the week before diagnosis, thoracic outlet syndrome (TOS) and upper extremity DVT with unilateral catheter as provoking factors.

### Outcomes

Patients were followed for 24 months from index VTE. Cancer diagnoses were obtained from individual medical records. The medical records cover the vast majority of primary care providers and hospitals in Stockholm county. We therefore anticipate to have included all cancer cases. Patients diagnosed with cancer at baseline (≤ 10 days after VTE) would not have benefitted from a screening program and were therefore excluded in the outcome analyses. No standardized screening program was applied, patients were screened according to attending physician’s clinical judgement. The RIETE score was computed retrospectively and did not affect clinical practice.

### RIETE score classifications

Patients were classified according to the RIETE score [9], classifying patients as having a high risk of occult cancer if the score was ≥ 3, and low risk of occult cancer if the score was ≤ 2. Points were given for male sex (+ 1p), age > 70 years (+ 2p), chronic lung disease (+ 1p), anemia (+ 2p, defined as hemoglobin < 130 g/l in men and < 120 g/l in women), elevated platelet count (+ 1p, defined as platelet count ≥ 350 × 10^9^/l), postoperative status (− 2p) and prior VTE (− 1p).

### Statistics

Standard deviations are reported for parametric data, and medians and interquartile ranges for non-parametric data. The student t test and chi^2^ test (or the Fisher exact test when appropriate) were used to compare continuous or categorical variables. Kaplan–Meier curves were constructed to illustrate time to new cancer diagnosis and calculate cumulative incidence, and were compared using the log-rank test. Baptista-Pike was used to obtain odds ratios (OR) with 95% (CI) for the outcome new cancer diagnosis during follow-up (i.e. 11 days–24 months after VTE) for the different sub-groups (provoked vs. unprovoked VTE and high vs. low risk according to the RIETE score). To further evaluate the RIETE score, we plotted a receiver operating characteristic (ROC) curve, and estimated the area under the curve and its 95% CI, presented as c-statistic. Complete case analysis was used. GraphPad Prism 7 (GraphPad Software, Inc., La Jolla, CA, USA) was used for all statistical analyses. A two-sided P < 0.05 was considered to be statistically significant.

## Results

**Fig. 1:**
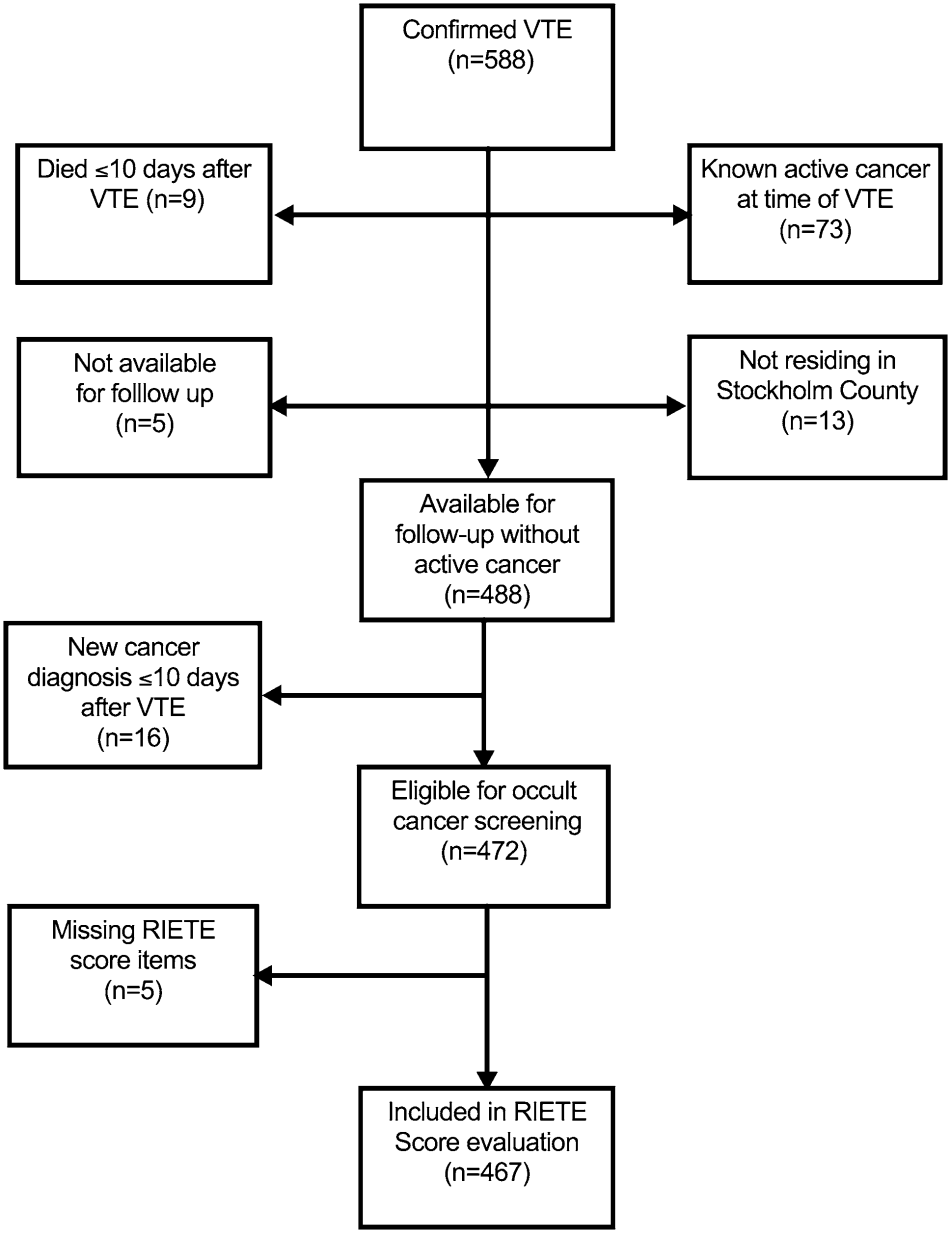
Flowchart of study patients

**Fig. 2:**
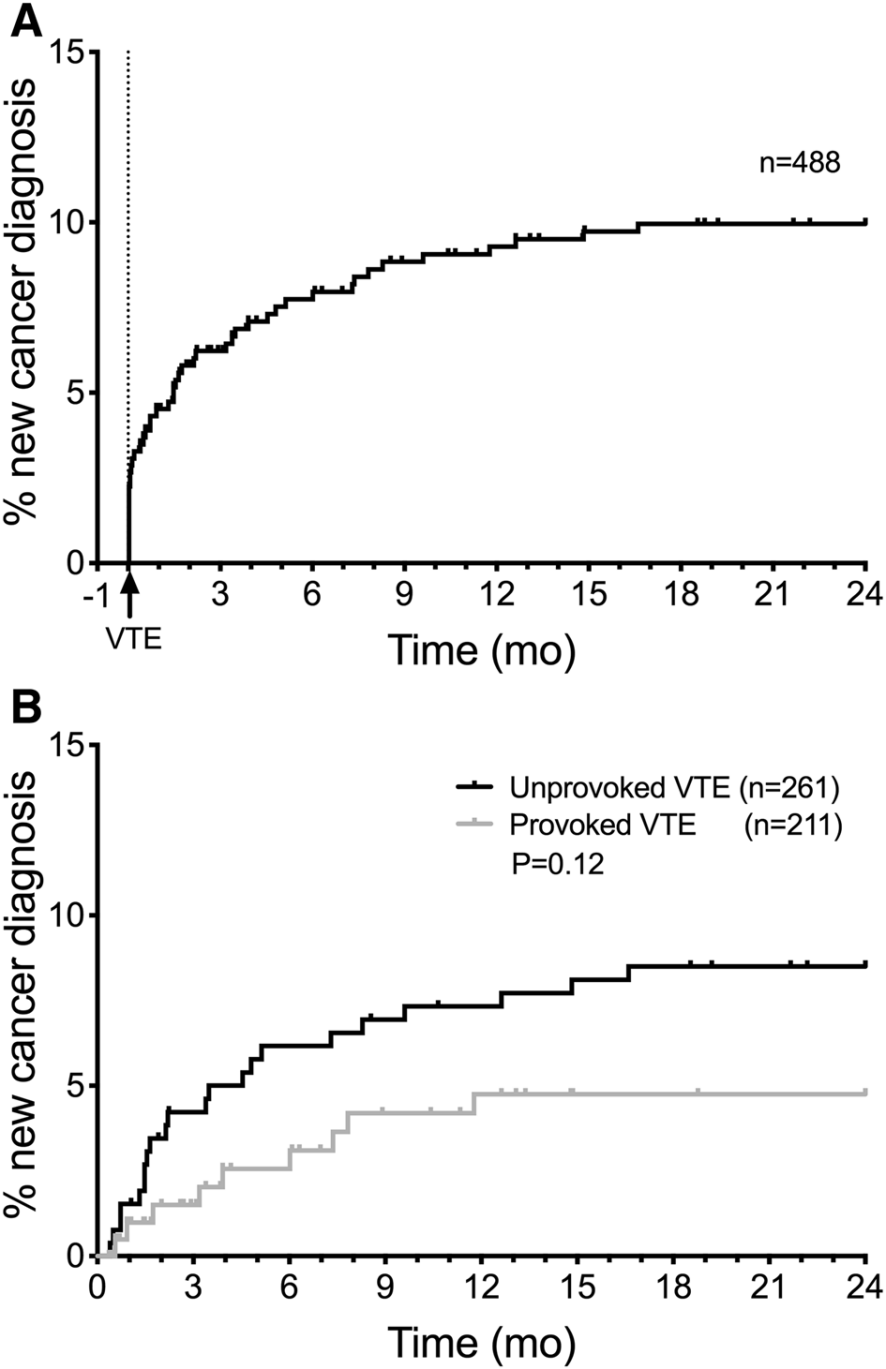
**a** Cumulative incidence of new cancer diagnosis within 2 years from VTE. New cancer diagnosis ≤ 10 days after VTE are included, patients who were lost to follow-up, died < 10 days of VTE diagnosis, not residing in Stockholm County or had a known active cancer at time of VTE are excluded. **b** Cumulative incidence of new cancer diagnosis within 24 months after unprovoked and provoked VTE in patients eligible for occult cancer screening (i.e. patients with new cancer diagnosis ≤ 10 days after VTE excluded)

A total of 588 patients were diagnosed with a confirmed VTE (Fig. 1). Patients with known active cancer at time of VTE (n = 73), that died ≤ 10 days after VTE (n = 9), that were not residing in Stockholm County (n = 13), or were not available for follow up (n = 5) were excluded from further analyses. Of the remaining 488 patients, 47 patients (9.6%) received a new cancer diagnosis during the 24 months following the VTE, of which 94% (44/47 patients) were diagnosed within 12 months (Fig. 2a). The cumulative incidence of cancer diagnosis was 7.7% at 6 months, 9.3% at 12 months and 10.0% at 24 months. The most common cancer sites among the cancers diagnosed were lung (17%), prostate (17%), ovarian (11%) and hematological malignancies (11%) (Supplementary Table S1). Four cases (8.5%) were relapses of a previously known, but not considered active, cancer.

### Patients eligible for occult cancer screening

Patients diagnosed with cancer at baseline (≤ 10 days after VTE, n = 16) were not considered eligible for occult cancer screening. A total of 472 patients were thus included in further outcome analyses. The median age in this group was 68 (IQR 53–78), and 258 patients (55%) were men. Among these patients, 261 (55%) had an unprovoked VTE, 234 (50%) presented with DVT, 197 (42%) with PE and 41 (8.7%) with concomitant DVT and PE. A flowchart of occult cancer screening in these patients is seen in Supplementary Figure S3. In all, 31 of these 472 patients (6.6%) received a new cancer diagnosis during the follow-up of 11 days–24 months after VTE. The cumulative incidence was high after both unprovoked (8.5%) and provoked VTE (4.8%), P = 0.12 (Fig. 2a).

Patients with prior cancer (OR 5.22 [95% CI 2.39–11.5]) and COPD (OR 4.00 [95% CI 1.55–9.50]) were more likely to receive a new cancer diagnosis during follow-up. A comparison of baseline characteristics for patients with no new cancer diagnosis during follow-up and patients with new cancer diagnosis during follow-up is seen in Table 1.

**Table 1:** Baseline characteristics of study patients eligible for occult cancer screening

	No active or new cancer diagnosis (n = 441)	Cancer diagnosis 11 days–24 months after VTE (n = 31)	OR (95% CI)	P value
Male sex, no. (%)	247 (56)	14 (45)	0.65 (0.30–1.31)	0.27
Age, median (IQR), year	68 (51–78)	71 (61–78)	–	0.095
BMI, median (IQR)	26.0 (23.5–28.8)	25.4 (21.9–28.3)	–	0.26
Prior cancer, no. (%)	42 (9.5)	11 (35.5)	5.22 (2.39–11.5)	< 0.001
Initial VTE presentation, no. (%)				
DVT	222 (50)	12 (39)	0.62 (0.30–1.30)	0.27
DVT + PE	38 (8.6)	3 (9.7)	1.14 (0.35–3.81)	0.75
PE	181 (41)	16 (52)	1.53 (0.75–3.18)	0.26
Risk factors for VTE^a^, no. (%)				
No provoking factor (unprovoked)	239 (54)	22 (71)	2.07 (0.92–4.67)	0.092
Recent surgery	51 (12)	1 (3.2)	0.25 (0.025–1.39)	0.23
Hospital stay	58 (20)	4 (13)	0.61 (0.23–1.65)	0.48
Bedridden/immobilized	53 (17)	4 (13)	0.72 (0.27–1.97)	0.80
Long distance travel	23 (5.2)	0	0 (0–2.17)	0.39
Estrogen use	27 (6.1)	2 (6.5)	1.06 (0.24–4.27)	> 0.99
Leg injury	40 (9.1)	1 (3.2)	0.33 (0.032–1.85)	0.50
Inflammatory disease	30 (6.8)	1 (3.2)	0.46 (0.043–2.60)	0.71
Prior VTE, no. (%)	99 (22)	8 (26)	1.20 (0.55–2.65)	0.66
Prior unprovoked VTE, no. (%)	58 (13)	4 (13)	0.98 (0.36–2.71)	> 0.99
Thrombophilia, no. (%)	33 (7.5)	1 (3.2)	0.41 (0.039–2.33)	0.72
COPD, no. (%)	30 (6.8)	7 (23)	4.00 (1.55–9.50)	0.007
Smoking, no. (%)	41 (9.3)	5 (16)	1.88 (0.75–4.85)	0.21
Prior smoking, no. (%)	181 (41)	18 (58)	1.99 (0.99–3.99)	0.089
Diabetes mellitus, no. (%)	36 (8.2)	4 (13)	1.67 (0.60–4.84)	0.32
Prior stroke/TIA, no. (%)	50 (11)	3 (9.7)	0.84 (0.26–2.73)	> 0.99
Prior MI, no. (%)	27 (6.1)	2 (6.5)	1.06 (0.24–4.27)	> 0.99
Heart failure, no. (%)	33 (7.5)	1 (3.2)	0.41 (0.039–2.33)	0.72
Platelet count^b^, median (IQR), 10^9^/l	215 (174–263)	225 (163–250)	–	0.67
Hemoglobin^c^, median (IQR), g/l	139 (127–149)	134 (127–148)	–	0.57
WBC count^d^, median (IQR), 10^9^/l	8.4 (7.1–10.3)	8.2 (6–9.8)	–	0.18

*CI* Confidence interval, *IQR* interquartile range, *BMI* body mass index, *VTE* venous thromboembolism, *DVT* deep vein thrombosis, *PE* pulmonary embolism, *COPD* chronic obstructive pulmonary disease, *TIA* transitory ischemic attack, *MI* myocardial infarction, *WBC* white blood cell

^a^The provoking factors pregnancy, cesarean section, DVT with unilateral catheter and thoracic outlet syndrome were present in less than 5 patients each and are not presented above

^b^Platelet count was unknown in five patients

^c^Hemoglobin levels were unknown in two patients

^d^WBC count was unknown in four patients

### RIETE score evaluation

**Fig. 3:**
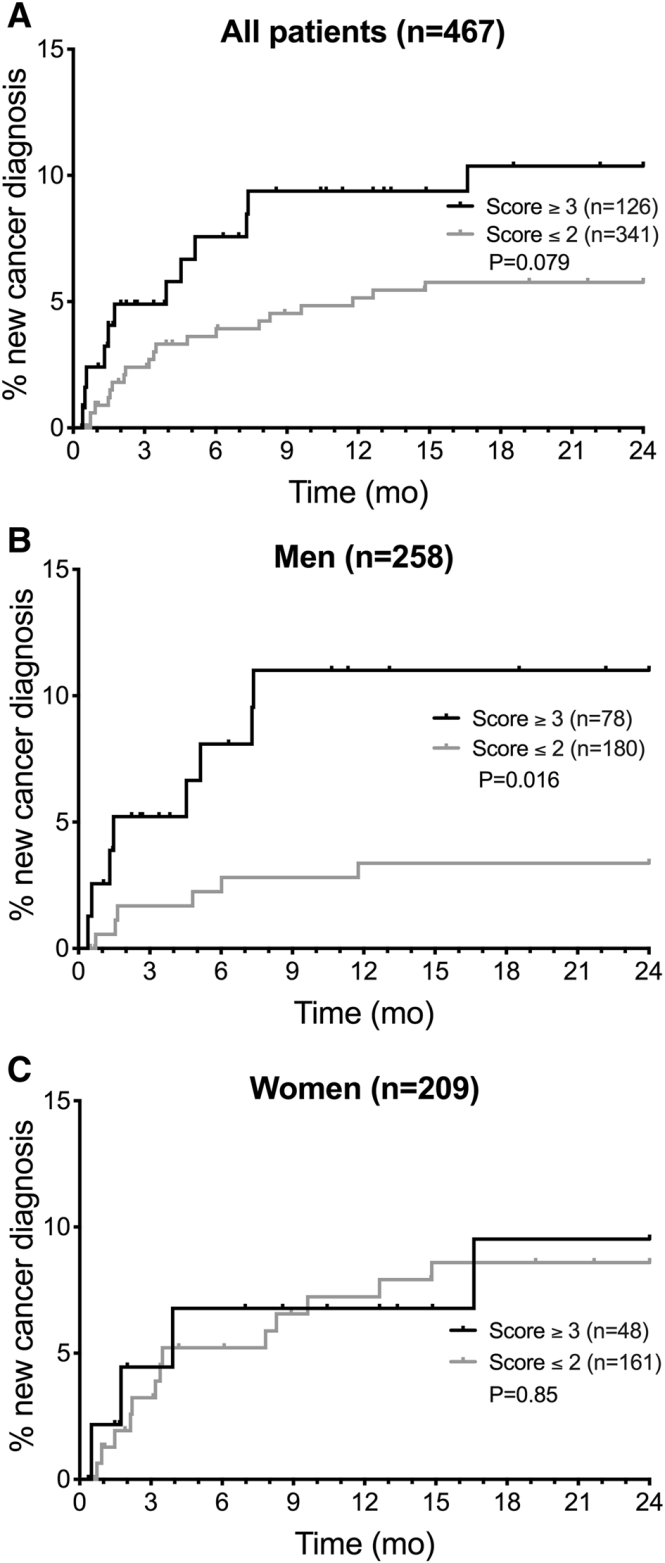
Cumulative incidence of new cancer diagnosis 11 days to 24 months after VTE. **a** Comparison of patients with a RIETE score ≥ 3 points vs ≤ 2 points. **b** Comparison of male patients with a RIETE score ≥ 3 points vs ≤ 2 points. **c** Comparison of female patients with a RIETE score ≥ 3 points vs ≤ 2 points

Platelet count and/or hemoglobin levels were missing in five of the 472 patients considered eligible for occult cancer screening. The performance of the RIETE score was therefore evaluated in 467 patients. Twenty-seven percent (126/467) of these patients were classified as having a high risk for an occult cancer according to the RIETE score (i.e. ≥ 3 points). The cumulative incidence of cancer during follow-up was 10.4% in the high-risk group, compared to 5.8% in the low risk group, P = 0.079 (Fig. 3a). A high RIETE score was not significantly associated with new cancer diagnosis (OR 1.78 [95% CI 0.85–3.63]) (Table 2). The sensitivity of the score was 0.39 (95% CI 0.24–0.56), the specificity 0.74 (95% CI 0.70–0.78), the negative predictive value 0.94 (95% CI 0.91–0.96) and the positive predictive value 0.095 (95% CI 0.055–0.16). The C-statistic for the RIETE score was 0.56 (95% CI 0.45–0.67).

**Table 2:** RIETE score items, according to cancer diagnosis 11 days–24 months after VTE

	No cancer diagnosis (n = 436)	Cancer diagnosis 11 days–24 months after VTE (n = 31)	OR (95% CI)	P value
RIETE ≥ 3p, no. (%)	114 (26)	12 (39)	1.78 (0.85–3.63)	0.14
Male sex (+ 1p), no. (%)	244 (56)	14 (45)	0.65 (0.30–1.31)	0.27
Age over 70 (+ 2p), no. (%)	182 (42)	18 (58)	1.93 (0.96–3.88)	0.091
COPD (+ 1p), no. (%)	30 (6.9)	7 (23)	3.95 (1.53–9.38)	0.0071
Anemia^a^ (+ 2p), no. (%)	86 (20)	7 (23)	1.19 (0.47–2.75)	0.65
Elevated platelets^b^ (+ 1p), no. (%)	36 (8.3)	1 (3.2)	0.37 (0.035–2.07)	0.50
Recent surgery (− 2p), no. (%)	49 (11)	1 (3.2)	0.26 (0.025–1.44)	0.23
Prior VTE (− 1p), no. (%)	96 (22)	8 (26)	1.23 (0.57–2.73)	0.66

*CI* Confidence interval, *COPD* chronic obstructive pulmonary disease, *VTE* venous thromboembolism

^a^Anemia was defined as hemoglobin < 130 g/l in men and < 120 g/l in women

^b^Elevated platelets were defined as platelet count ≥ 350 × 10^9^/l

The score performed better in men than in women (Fig. 3b, c) with an OR of 3.31 (95% CI 1.07–9.99) vs. 1.04 (95% CI 0.30–2.83).

## Discussion

In this retrospective cohort study, the incidence of new cancer diagnosis was relatively high after both unprovoked and provoked VTE. The RIETE score was not able to identify patients with a high risk for occult cancer, which was mainly due to a poor performance in women.

The incidence of occult cancer was higher in our cohort compared to several recent studies [4–9, 14]. The median age was also higher in our cohort compared to many of these studies [6, 8, 9], conceivably due to the unselected nature of our clinical practice-based approach, which could explain the higher incidence of new cancer diagnosis. Indeed, age is a component of the RIETE score. In line with this, other studies with higher mean/median age tend to have higher incidences of new cancer diagnoses, such as Sandén et al [4], Ferreyro et al [15] and Prandoni et al [7], with 9.6%, 9.2% and 9.2% new cancer diagnoses, respectively.

Consistent with prior studies [4, 16], the vast majority (94%, 44/47) of new cancer cases in our cohort were diagnosed within 12 months of VTE diagnosis. The RIETE score was developed to identify patients with an elevated risk of new cancer diagnosis within 24 months, and many previous studies have employed a 24-month follow-up. We therefore decided a priori to follow study patients for 24 months, but a 12-month follow-up appears to be sufficient in designing future studies on occult cancer in VTE patients. A longer follow up also conveys a risk of including cancer diagnoses not associated with the index VTE.

Patients with provoked VTE are considered to have a low risk for occult cancer, and recent guidelines recommend cancer screening in unprovoked VTE only [17]. In contrast to this view, we found the rate of occult cancer to be relatively high after both unprovoked and provoked VTE. As with all thrombosis, one must overcome a threshold. An accumulation of prothrombotic factors (i.e. a known provocation such as hospital stay or long distance travel *and* an occult cancer) may thus overcome the threshold and trigger a VTE. Indeed, the observed elevated risk of occult cancer in patients presenting with provoked VTE is corroborated by other recent studies [9, 12] and may challenge the current praxis of limiting cancer screening to patients with unprovoked VTE. There was, however, a trend towards a higher rate of new cancer diagnosis in patients with unprovoked VTE in our cohort, and we cannot rule out that the lack of statistical significance between the rate of new cancer diagnosis between patients with provoked and unprovoked VTE may be due to a small sample size.

Many previous studies on occult cancer in VTE patients exclude patients with prior cancer at any time [9, 15] or within 5 years prior to VTE [8]. The threshold for occult cancer screening in patients with VTE could arguably be lower in patients with prior cancer. Our experience is not, however, that an extensive screening is performed in all VTE patients with a prior but not active cancer, urging for risk prediction scores to be applied to these patients. All variables associated with an increased risk should be candidates for risk prediction scores to aid in clinical decision making, similar to the inclusion of the variable prior stroke in CHA_2_DS_2_-VASc [18], a risk score for estimating the risk of stroke in patients with atrial fibrillation. Patients with prior cancer are common in a routine clinical setting, and 53 of the patients (11%) eligible for occult cancer screening in our cohort had a prior, but not active, cancer diagnosis. We included these patients in our study and, consistent with a previous study [12], prior cancer was the variable that showed the strongest association to new cancer diagnosis during follow-up. Notably, only four out of the 47 cancer cases were relapses of a previously known but not active cancer, with the remaining cancer diagnoses comprising new primary tumors. These results may justify the inclusion of patients with prior, but not active, cancer diagnoses in future studies of occult cancer in VTE patients.

The RIETE score was not able to identify a subgroup of patients with a high risk of occult cancer, contrary to prior validations [10–12]. When applied only to men, the score was able to identify a subgroup of patients with a high risk of occult cancer, but the performance was poor in the whole group. The low discriminative capacity of the RIETE score was mainly due to a poor performance in women, consistent with recently published data [19]. Considering that almost half of the VTE patients were women, this discrepancy is concerning. We believe that risk scores, such as the RIETE score, should include both genders. The possible addition of blood biomarkers to clinical characteristics similar to bleeding risk estimation [20] could potentially yield a better, and perhaps more gender equal score. Ongoing studies are therefore investigating the additive value of various biomarkers in detecting occult cancer in patients presenting with VTE (ClinicalTrials.gov; NCT02739867 and NCT03781531).

The main strength of the study is the unselected patient population resulting from the database including all patients treated for VTE, as opposed to prospective studies with possible inclusion bias. Ninety-six percent of all patients eligible for cancer screening in the database were included in the study. However, our study is limited by the retrospective nature of the study and the relatively small sample size.

Taken together, our results show a relatively high rate of new cancer diagnoses among patients with both unprovoked and provoked VTE. Although the RIETE score was able to identify men with a high risk of occult cancer in our cohort of VTE patients, the score performed poorly in women. Further risk score models are therefore warranted.

## Electronic supplementary material

Below is the link to the electronic supplementary material.

Supplementary material 1 (DOCX 461 KB)
