# Supplementary material for: A clinical practice-based evaluation of the RIETE score in predicting occult cancer in patients with venous thromboembolism
Source: J Thromb Thrombolysis. 2019 Feb 9;48(1):111–8. doi: 10.1007/s11239-019-01822-z (PMC6556156; doi:10.1007/s11239-019-01822-z)
Supplement: Supplementary file 1 — Supplementary material 1 (DOCX 461 KB) [file 11239_2019_1822_MOESM1_ESM.docx]

A clinical practice-based evaluation of the RIETE score in predicting occult cancer in patients with venous thromboembolism

Axel Rosell^a^, Staffan Lundström^b,c^, Nigel Mackman^d,e^, Håkan Wallén^f^, Charlotte Thålin^a^

a Department of Clinical Sciences, Danderyd Hospital, Division of Internal Medicine, Karolinska Institutet, Stockholm, Sweden

b Palliative Care Services and R&D-unit, Stockholms Sjukhem Foundation, Stockholm, Sweden

c Department of Oncology-Pathology, Karolinska Institutet, Stockholm, Sweden

d Department of Medicine, Division of Hematology and Oncology, Thrombosis and Hemostasis Program, University of North Carolina at Chapel Hill, Chapel Hill, NC, USA

e Department of Medicine, University of North Carolina at Chapel Hill, Chapel Hill, NC, USA

f Department of Clinical Sciences, Danderyd Hospital, Division of Cardiovascular Medicine, Karolinska Institutet, Stockholm, Sweden

**Corresponding author:**

Charlotte Thålin, MD, PhD.,

Email address: charlotte.thalin@ki.se

**Supplementary Table S1**

*Sites of new cancer diagnosis within 24 months of VTE.*

| Site | Cancer diagnosis within 24 months of VTE  (n=47) |
| --- | --- |
| Lung, No. (%) | 8 (17) |
| Prostate, No. (%) | 8 (17) |
| Colorectal, No. (%) | 4 (8.5) |
| Bladder, No. (%) | 2 (4.3) |
| Hematologic (incl. Lymphoma), No. (%) | 5 (11) |
| Kidney, No. (%) | 2 (4.3) |
| Pancreas, No. (%) | 4 (8.5) |
| Breast, No. (%) | 4 (8.5) |
| Ovarian, No. (%) | 5 (11) |
| Uterus, No. (%) | 1 (2.1) |
| Other^a^, No. (%) | 2 (4.3) |
| Unknown origin, No. (%) | 2 (4.3) |

VTE, Venous thromboembolism. ^a^Tonsil cancer and vulvar cancer.

**Supplementary Table S2**

Baseline characteristics of patients with cancer diagnosis ≤ 10 days after VTE and known active cancer at time of VTE.

|  | Cancer diagnosis ≤ 10 d after VTE (n=16) | Known active cancer at time of VTE (n=73) |
| --- | --- | --- |
| Male sex, No. (%) | 8 (50) | 42 (58) |
| Age, median (IQR), y | 71.5  (63.8-81.8) | 74  (67.5-80) |
| BMI, median (IQR) | 24.4  (23.0-29.4) | 24.7  (21.6-29.3) |
| Prior cancer, No. (%) | 8 (50) | - |
| Initial VTE presentation, No. (%) |  |  |
| *DVT* | 4 (25) | 36 (49) |
| *DVT+PE* | 4 (25) | 6 (8.2) |
| *PE* | 8 (50) | 31 (44) |
| Risk factors for VTE^a^, No. (%) |  |  |
| *No provoking factor (unprovoked)* | 11 (69) | 0 (0) |
| *Recent surgery* | 1 (6.3) | 14 (19) |
| *Hospital stay* | 3 (19) | 28 (38) |
| *Bedridden/immobilized* | 0 | 11 (15) |
| *Long distance travel* | 1 (6.3) | 0 |
| *Estrogen use* | 1 (6.3) | 1 (1.4) |
| *Leg injury* | 0 | 2 (2.7) |
| *Inflammatory disease* | 1 (6.3) | 9 (12) |
| Prior VTE, No. (%) | 1 (6.3) | 14 (19) |
| Prior unprovoked VTE, No. (%) | 0 | 5 (6.9) |
| Thrombophilia, No. (%) | 0 | 4 (5.5) |
| COPD, No. (%) | 1 (6.3) | 5 (6.9) |
| Smoking, No. (%) | 2 (13) | 2 (2.7) |
| Prior smoking, No. (%) | 6 (38) | 35 (48) |
| Diabetes mellitus, No. (%) | 3 (19) | 9 (12) |
| Prior stroke/TIA, No. (%) | 3 (19) | 6 (8.2) |
| Prior MI, No. (%) | 3 (19) | 4 (5.5) |
| Heart failure, No. (%) | 3 (19) | 4 (5.5) |
| Platelet count^b^, median (IQR), 10^9/L | 222  (176-283) | 208.5  (159-279) |
| Hemoglobin^c^, median (IQR), g/L | 129  (110-141) | 120  (104-130) |
| WBC count^d^, median (IQR), 10^9/L | 10.4  (10-15) | 7.9  (6.2-13) |

CI, Confidence interval; IQR, Interquartile range; BMI, Body mass index; VTE, Venous thromboembolism; DVT, Deep vein thrombosis; PE, Pulmonary embolism; COPD, Chronic obstructive pulmonary disease; TIA, transitory ischemic attack; MI, myocardial infarction; WBC, White blood cell.

^a^The provoking factors pregnancy, cesarean section, DVT with unilateral catheter and thoracic outlet syndrome were present in less than 5 patients each and are not presented above. ^b^Platelet count was unknown in one patient. ^c^Hemoglobin levels were unknown in one patient. ^d^WBC count was unknown in two patients.

**
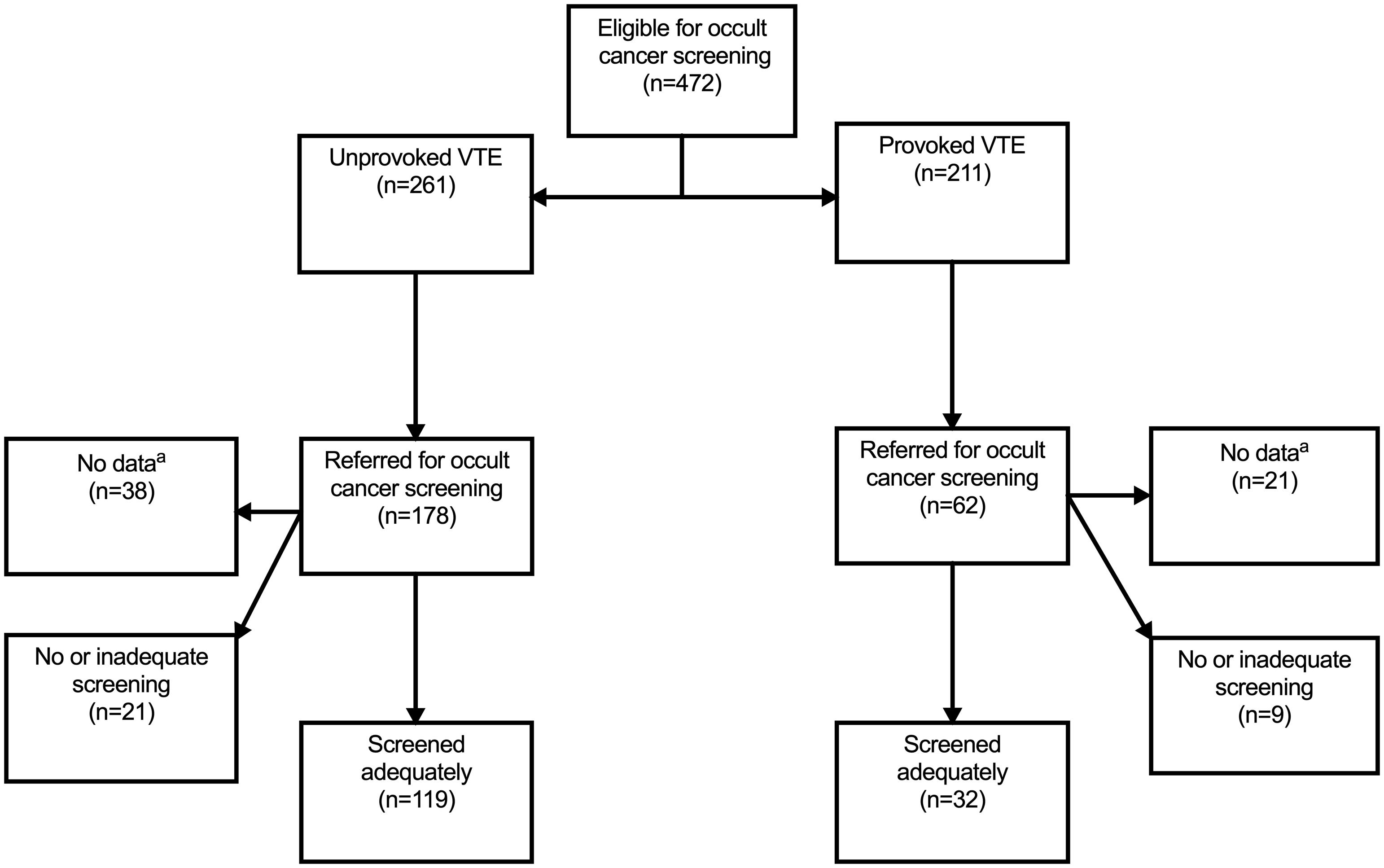
**

**Supplementary Figure S3.** A flowchart of occult cancer screening. ^a^Some providers of primary care used a different software for handling patient records, and they were therefore not available for examination.
